# The Unculturables: targeted isolation of bacterial species associated with canine periodontal health or disease from dental plaque

**DOI:** 10.1186/1471-2180-14-196

**Published:** 2014-08-01

**Authors:** Ian J Davis, Christopher Bull, Alexander Horsfall, Ian Morley, Stephen Harris

**Affiliations:** 1 The WALTHAM® Centre for Pet Nutrition, Mars Petcare, Leicestershire, UK; 2 WALTHAM® Centre for Pet Nutrition, Mars Petcare UK, Waltham on the Wolds, Melton Mowbray LE14 4RT, UK

**Keywords:** Periodontal disease, Periodontitis, Uncultivable, Quantitative PCR, QPCR, Bacterial isolation, Helper strain, Unculturable

## Abstract

**Background:**

The current inability to culture the entirety of observed bacteria is well known and with the advent of ever more powerful molecular tools, that can survey bacterial communities at previously unattainable depth, the gap in our capacity to culture and define all of these species increases exponentially. This gap has essentially become the rate limiting step in determining how the knowledge of which species are present in a sample can be applied to understand the role of these species in an ecosystem or disease process. A case in point is periodontal disease, which is the most widespread oral disease in dogs. If untreated the disease results in significant pain, eventual loss of the dentition and potentially an increased risk of systemic diseases. Previous molecular based studies have identified the bacterial species associated with periodontal disease in dogs; however without cultured strains from many of these species it has not been possible to study whether they play a role in the disease process.

**Results:**

Using a quantitative polymerase chain reaction (qPCR) directed approach a range of microbiological media were screened and optimized to enrich for previously uncultivated target species. A systematic screening methodology was then employed to isolate the species of interest. In cases where the target species were not cultivable in isolation, helper strains grown underneath a nitrocellulose membrane were used to provide the necessary growth factors. This guided media optimization approach enabled the purification of 14 species, 8 of which we had previously been unable to cultivate in isolation. It is also applicable to the targeted isolation of isolates from species that have previously been cultured (for example to study intra-species variation) as demonstrated by the successful isolation of 6 targeted isolates of already cultured species.

**Conclusions:**

To our knowledge this is the first time this combination of qPCR guided media optimization, strategic screening and helper strain support has been used successfully to isolate previously uncultured bacteria. This approach can be applied to any uncultured bacterial species where knowledge of their nutritional requirements or low relative abundance impedes their isolation.

## Background

As far back as 1911, the inability to culture the entirety of observable bacteria (at the time via microscope) was well understood [1]. With the advent of next-generation molecular tools to identify bacterial species, the number of phyla currently stands in excess of 85 (versus 11 in 1987), the majority of which are unculturable [2,3]. “Unculturable” in this context meaning the inability to grow on artificial media *in vitro*. Metagenomics now offers us the chance to gain an insight into the genetic potential of these unculturable species; however the inability to culture certain bacterial species in the laboratory makes it impossible to fully characterize and understand their role in an ecosystem or disease process. A case in point is periodontal disease in humans, where it is generally accepted that bacteria are the aetiological agent of disease. Despite extensive research, is not yet clear how and which specific bacterial species initiate the disease process [4]. This is a greater issue in dogs where periodontal disease is the most widespread oral disease. Studies have demonstrated that between 44% and 63.6% of dogs are affected by the disease [5-8].Variation in prevalence estimates are likely to be due to the different age and breed compositions of the study groups and the criteria used to establish diagnosis of periodontal disease.

To better understand the role of bacteria in initiating periodontal disease in dogs, identification and culture of the relevant bacterial species is required. Our previous studies have described the diversity of the canine oral microbiome and also the association of specific bacterial species with health, gingivitis or mild periodontal disease in dogs [9,10]. These studies were based on molecular screening of bacterial DNA in plaque taken from client owned dogs. Our culture based investigations using standard media and microbiological approaches have isolated many, but not all, examples of the key species in the canine oral microbiome [11]. Based on the phylogenetic trees of the canine oral microbiome described by Dewhirst *et al.*, it is evident that the majority of missing species are closely related including groups of species within the Clostridiales, Peptostreptococcaceae, Lachnospiracea, Porphyromonads and Bacteroidetes [9]. This suggests that the growth conditions used in previous studies were not optimal to support the growth of species within these clades, either due to missing nutrients, toxicity of the media or a mutualistic dependence on other species.

There are numerous reports of isolation strategies for culturing recalcitrant bacteria from specific habitats [12,13]. In order to isolate species found in coastal subsurface sediments Kopke *et al*. mimicked the environmental conditions *in vitro* by producing media with substrate gradients similar to those found in coastal sediment ecologies [13]. Ferrari *et al.,* mimicked both environmental nutrient conditions and provided mutualistic dependencies through the use of filter membranes that separated non-sterile soil slurry from the species they were attempting to isolate. This provided the required soil metabolites along with the secondary metabolites and signaling compounds produced by other symbiotic species [14]. An alternative approach is to monitor how effective a chosen medium is in enriching for selected species. Via DGGE profiles Tian *et al.* demonstrated that an optimized medium supported a more diverse array of species than standard media; although this medium was not optimized for specific target species [14].

Based on a combination of these approaches the objective of this study was to formulate a systematic screening system that would enable the identification of microbiological media/growth conditions that support the growth of specific previously uncultivated bacteria which represented gaps in the current canine oral microbiome phylogenetic tree [9]. Whilst in the case of this study the aim was to isolate specific canine oral bacterial species, this approach is also applicable for the isolation of previously unculturable target bacteria from a wide range of other habitats.

## Methods

### Sample isolation and preparation

Plaque samples were taken from dogs under anesthesia for plaque collection for a separate ongoing oral care trial which was approved by the WALTHAM^®^ Centre for Pet Nutrition ethical review committee under licensed authority in accordance with the UK Animals (Scientific Procedures) Act 1986. Plaque was sampled from the following teeth (lower jaw: incisors, 01–03, premolars 05–07 & molars 10 and upper jaw: incisors 01–02, premolars 05–06 & molars 10). Each dog was given a premedication of 0.02 mg/kg acepromazine (ACP 2 mg/ml) and 0.02 mg/kg buprenorphine (Vetergesic 0.3 mg/ml) intramuscularly, then induced with 0.4 mg/kg propofol (Rapinovet 10 mg/ml) given intravenously, and maintained on 2% inhalation isoflurane. Initially, supra-gingival and gingival margin plaque and calculus were removed using a Gracey curette to prevent contamination of the sub-gingival sample. A periodontal probe was then inserted under the gingival margin and swept along the tooth surface. Sub gingival plaque samples were taken from individual teeth, put immediately into pre-reduced 300 μl TE buffer (Sigma Adrich, UK) and immediately processed.

### Screen for optimal media

**Figure 1:**
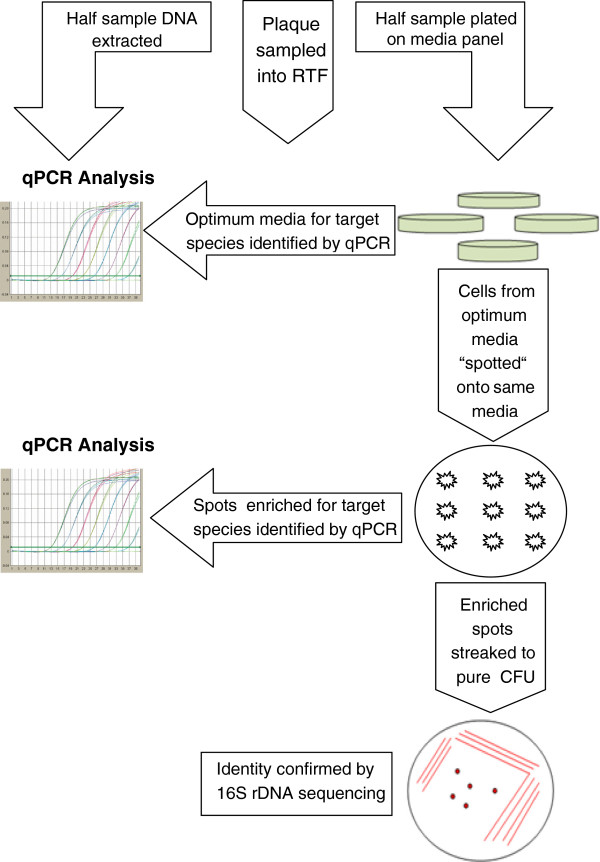
**An overview of the screening and isolation process used to isolate target bacterial species.** RTF denotes reduced transport fluid and qPCR describes quantitative PCR.

An overview of the approach is shown in Figure 1. Plaque samples were homogenized by vortex followed by repeated pipetting then split into two 150 μl aliquots. To one aliquot, 150 μl of reduced transport fluid (RTF) (9 mg/ml, (NH_4_)_2_SO_4_; 9 mg/ml, NaCl; 4.5 mg/ml, K_2_HPO_4_; 4.5 mg/ml, KH_2_PO_4_; 4 mg/ml, Na_2_CO_3_; 3.8 mg/ml, EDTA; 2 mg/ml Dithiothreitol; 1.8 mg/ml, MgSO_4_ (heptahydrate) and 0.1% Resazurin) was added to further lower the oxygen concentration within the sample before it was serially diluted (neat down to 10^-5^) and the dilutions plated on a panel of various microbiological media that had been pre-reduced overnight (Table 1). The remaining half was sent for Q-PCR analysis to quantify the levels of target species in the plaque sample (described below).

**Table 1:** Media used in the screening panel

**Media**	**Target bacteria**
Columbia blood agar (CBA) (Oxoid, UK)	Aerobes or Anaerobes when supplemented with H + M
Fastidious anaerobe agar (FAA) + H + M (LabM, UK)	Anaerobes
Tryptone soya agar (TSA) (Oxoid, UK)	Aerobes or Anaerobes when supplemented with H + M
Heart infusion agar (HIA) (Oxoid, UK)	Anaerobes
Artificial saliva agar (ASA) (see text) + H + M	Anaerobes
Blood agar base no. 2 (Oxoid, UK)	Aerobes or Anaerobes when supplemented with H + M
All of the above + kanamycin (75 μg/ml; Sigma-Aldrich, UK)	Gram-negative bacteria
All of the above + Phenylethyl alcohol (PEA) (0.25% vol: final vol; Sigma-Aldrich, UK)	Gram-positive cocci

All media were supplemented with 5% horse blood. H + M = hemin (5 μg/ml; Sigma-Aldrich, UK) and menadione (0.5 μg/ml; Sigma-Aldrich, UK).

Dependent on the target species, plates were incubated for 7 days at 38°C in aerobic, microaerophilic (5% O_2_, 10% Co_2_ in nitrogen) or anaerobic (85% N_2_, 10% CO_2_, and 5% H_2_) conditions in a MG1000 Anaerobic Work Station (Don Whitley Scientific Ltd., Shipley, United Kingdom). Media were made up as per supplier’s instructions with the exception of the artificial saliva agar which was modified to better reflect canine saliva (In 1 liter: 1 g Lab lemco powder; 2 g Yeast extract; 5 g Proteose peptone, 2.5 g Hog gastric mucin; 0.82 g NaCl; 0.1 g CaCl_2_; 1.5 g KCl; 0.11 g MgCl_2_ & 10 g Agar no1). Subsequently, for each media the lowest dilution plate that had discreet mixed or single species macro colonies (as opposed to confluent growth) was washed with 500 μl RTF to remove the bacterial cells. Half of this was re-plated onto the same media (for these purposes termed the template plate) and half subjected to qPCR analysis to identify which media best enriched for the target species of interest.

### Q-PCR Screens

DNA was extracted from each sample using Masterpure™ Gram Positive DNA Purification Kit (Epicentre MGP04100) following the manufacturer’s protocol except for an 18 hour lysozyme incubation phase. The resulting DNA was re-suspended in 70 μl TE Buffer. Bacterial samples from neat plate scrapes were diluted 2:3 in TE buffer (final volume of 150 μl) to avoid overloading the DNA extraction.

The bacterial DNA was analysed using Q-PCR on an ABI7900 HT fast real time PCR instrument using Taqman FAM-MGB labelled assays (see Table 2 for primer/probe sequences) with the assays designed either in-house or by Primer Design Ltd (Southampton, UK). To reduce the risk of false positives, assays were validated for specificity against more than 400 16S rRNA clones, representing all of the taxa currently identified in the canine oral microbiome [9].

**Table 2:** DNA sequence of oligonucleotide primer and probes sets used in qPCR screens for target species

**Assay**	**Target**	**Forward primer**	**Probe**	**Reverse primer**
COT005	*Peptostreptococcaceae* sp. COT-005	CGTAACCGAGGAAATTTTTCGA	TGGAATCAGTTACGTTTAGTG	GCAGGTTGCCCACGTGTT
COT033	*Peptostreptococcus sp.* COT-033	CGCGGTTGTGCTTAGTATTGAG	CACAACTGAGCGGCGG	TCCATGTGTATAGGGCAGGTTACC
COT016	*Neisseria animaloris* COT-016	AACTGTCCGAAAGGATGGCTAA	ATATTCTCTGAGGAGGAAAG	CGCAAGGCCCGAAGGT
COT192	*Porphyromonas* sp. IJD1952	CTTGCCTGATAGAAAGGGATAACC	TGAAAGTCGGACTAATAC	TCATGCAATAACCCAAGACCATA
COT108	*Porphyromonas canoris* COT-108	ATGGCGACCGGCGGAT	CCCCTCTGACAGGTAAGTTGCATACGC	CTTGAAATACCATGCAGYATCTCAAG
COT052	*Porphyromonas gulae II* COT-052	GTTGAAAGACGGACTAATACC	CCTTGCCCGRTCATGCAACCAAGCAAG	CATGCCTATCTTACAGCTATAAAT
COT080	*Pasteurellaceae [G-2] sp.* COT-080	CCTTCGGGTTGTAAAGTTCTT	AAGGTATCAACTWTAATAGAGTTGGTAAWDGACGTTAT	TGCTGGCACGGAGTTAG
COT084	*Odoribacter denticanis* COT-084	GGGTAACAGGCGGTAGCAATAC	ATGCAATCTACCTTTTACC	AAAGAAATGCATCGGGTATTAATCC
COT107	*Globicatella* sp. COT-107	CGGAAGGAGAACTTGTTCTTTGGA	TGTTACTCACCCGTGCGCCACT	GGTATTAGCACTCGTTTCCCAGT
COT036	*Lachnospiraceae* sp. COT-036	GAAGCRCGGGAAGCGGAAGT	CGTTACTCACCCGTCCGCCACT	CTTTTCCCTCYGTATCATGCGATAC
COT044	*Peptococcus* sp. COT-044	CGCATAATATCTCTTTATYGCATGATAG	CTAAACGACAGCDCTAAGGCCGTCTTT	TACTGATCGTCGCCTTGGT
COT064	*Filifactor* sp. COT-064	GGTGCGTAACGTGTGGGTAA	CCTTTGTCATGGGAATAA	TTCGGTATTAGCTGCTCTTTCAAG
COT227	*Peptostreptococcus* sp. COT-227	GCGACTGATTTGATGCTTGC	CACCCGTCCGCCGCTCAACTTTCAT	AACTTTTCAGTATGTTATCCATGTGTA
COT388	*Clostridiales* sp. COT-388	GGAAGAAGACTTCGGTCAACGGA	CGTTACTCACCCGTCCGCCACT	CATTTGGATGCCCATTCGGTATG
COT029	Tissierella sp. COT-029	GAAGAACCTGCCTTTCACATAGGA	CGGGATTAATACCAAATGA	CCCCAAAAACATGCGATCTC
COT306	*Chloroflexi* sp. COT-306	GAACGGGTGCAGCGATGT	TGTTACTCACCCGTGCGCCACT	TAATCTGAGACAGCTTATGCGGT
COT280	*Conchiformibius steedae* COT-280	GGGATAACTTGCCGAAAGGTAA	ACCTCGCGTTATCCGAGCGGCC	TAGGCTTTTACCCCACCAACT
Uni B	All genus	CTCCTACGGGAGGCAGCAG	CCAGCAGCCGCGGT	GGACTACCAGGGTATCTAATCCTGTT
Uni B		CCTGCGGGAGGCAGC		TACCGGGGTATCTAATCCCGTT
Uni B		CTTCTACGGAAGGCAGCAGTAG		ACTACCAGGGTATCTAGTCCTGTTCG
Uni B		CTTCTACGGGAGGCAGCAG		GACTACCAGGGTGTCTAATCCTGTT
Uni B		CGACGGGAGGCAGCAG		GACTACTAGGGTATCTAATCCTGTTTGC
Uni B		CTACGGGAGGCGGCAG		GACTACCAAGGTATCTAATCCTGTTTGC
Uni B				GACTACCAGGGAATCTAATCCTGTTT
Uni B				GACTATCAGGGTACCTAATCCTGTTTG
Uni B				GACTACCGGAGTATCTAATTCCGTTC
Uni B				GACTACCAGGGTATCTAATCCCGTT
Uni B				CGGGGCATCTAATCCCGT

In order to determine the proportion of the target species 16S rDNA in a sample the UniB assay was used to estimate the total number of 16S rDNA molecules in each reaction.

Each sample was run in 10 μl reactions in each assay in duplicate using Taqman gene expression master mix (Applied Biosystems 4369016). Each assay had a final concentration of 900nM of each primer and 250 nM of each probe per QPCR reaction. The universal primer set consisted of 17 primers (6 forward primers and 11 reverse primers) and one probe to maximise amplification of the 16S locus from all known canine oral taxa species. The resulting data were averaged between the duplicates and then the proportions of each species were calculated against the universal assay primer/probe set (UniB) values, UniB amplification being a measure of all species within each sample.

### Isolation of pure cultures

**Figure 2:**
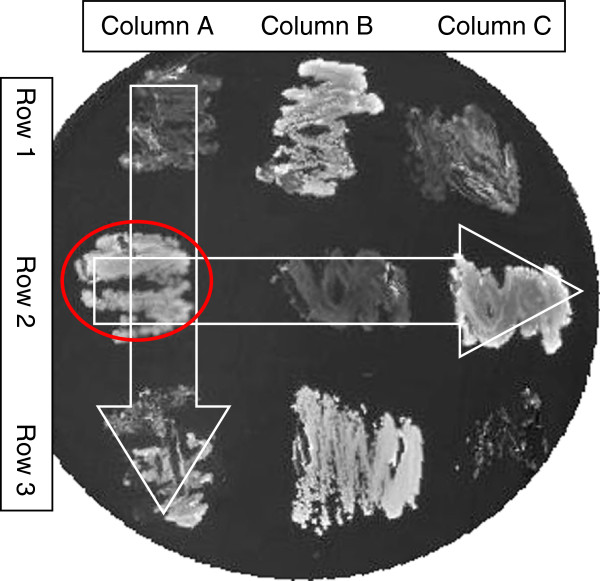
**A simplified example of the pooling strategy used to screen multiple mixed species colonies with the minimum number of qPCRs.** White arrows show how colonies were pooled by columns and by rows. If the mixed colony circled in red contains a target species a lower cQ will be observed in qPCR reactions from pools A and 2.

Where a medium was associated with relatively high levels of a target species (>5% of 16S rDNA present in the sample based on UniB primers), mixed colonies were taken from the template plate and spotted onto media of the same type in a grid format, incubated for 7 days prior to further Q-PCR analysis. A grid system of “spots” (mixed species colonies) was established to increase throughput. Cells from each of the mixed colonies in a column were pooled in 150 μl TE buffer using a 10 μl loop, giving a single sample per column. The same process was performed for spots in each row giving a single sample per row (Figure 2). Each pool was subjected to Q-PCR analysis with probes specific to the target species. The coordinates of a colony enriched for the target species were identifiable by cross referencing positive row pools against positive column pools. For example, if the red circled colony in Figure 2 contains the target species then both column pool A and row pool 2 will return positive Q-PCR data. Throughput was increased by using larger plates with more rows and columns; for example a 5 by 5 grid enabled screening of 25 colonies with 10 Q-PCR reactions.

Spots enriched for the target species were then re-streaked. If the re-streak resulted in mixed colonies these were screened via a further round of Q-PCR and the mixed colonies containing the target species re-streaked further. If single colonies could not be achieved the target enriched spots were re-streaked onto a helper strain (see below). Once individual colonies were achieved they were confirmed by 16S rDNA sequencing. Isolates with ≥98.5% shared identity with full length 16S rDNA sequence from the target species were considered examples of the target species.

### Use of helper strains

In the case of some mixed cultures where the target species was not cultivable in isolation on test media, *Propionibacterium acnes* was used as a helper strain. *P. acnes* was grown as a lawn on heart infusion agar for 48 h at 37°C under anaerobic conditions. A 0.45 μm nitrocellulose filter (RPN82E Hybond™ C-extra, GE Heathcare, UK ) was then placed on top of the colonies and the mixed culture enriched for the target species streaked on to the filter. Plates were incubated under the same conditions until single colonies were observed (usually within 7 days).

## Results and discussion

To our knowledge this is the first time this approach of supervised media optimization combined with a strategic screening process has successfully been used to isolate previously uncultured bacterial species. The key strength of this approach is the use of qPCR to estimate relative proportions of target species on any given media and thus make informed decisions on which media to proceed with. This approach is a progression of the work of Tian et al., [15] who used DGGE to identify the media that best supported the growth of complex and diverse bacterial communities as a whole. In that technique, the use of DGGE enabled the diversity of community profiles to be visualized and compared. Here, qPCR provides an extra level of granularity since proportions of specific species can be observed. Examples of qPCR data demonstrating the drop in Ct values as the relative prevalence of three target species (COT-064, COT-107 & COT-227) increased through the screening process is given in the supplementary data (Additional files 1 and 2) along with the 16S rRNA sequence generated to confirm their identity (Additional file 3). It should be noted that the relative proportions of the target species versus all species in the mixed colonies (calculated against the universal UniB probe set) are estimates and that for each probe set differences in amplification efficiency should be accounted for. However as evidenced in Supplementary data 1 the estimates provide a clear guide of which media, conditions and colonies to prioritize for subsequent rounds of screening. The work of Dewhirst [9] has been fundamental to the success of this approach as it has provided a relevant 16S rRNA clone database that has been used to extensively validate the specificity of the qPCR assays. In the absence of such a clone library, this validation would still have been possible through *in silico* methods*.* Alternatively, as the next generation of bench top sequencing technologies and associated bioinformatics methods become more available and affordable they could take the place of qPCR in this method.

In this study, a total of 11 species were targeted for isolation (Table 3) as they represented either species shown previously to be associated with periodontal health and disease or key gaps in representative isolates from the current canine oral microbiome phylogenetic tree [9,10]. An additional six species were targeted as additional isolates of species that had previously been cultivated to determine inter-species variability. Our methodology allowed us to culture isolates for 8 of the 11 previously uncultivated species and all 6 of the alternate isolate species.

**Table 3:** Bacterial species targeted using this approach and the media required to isolate them

**Canine oral taxon**	**Species**	**Media**	**Atmosphere**	**Disease association**
COT-005	*Peptostreptococcaceae XI [G-1] sp.*	HIA	Anaerobic	PD
COT-084	*Odoribacter denticanis*†	HIA	Anaerobic	PD
COT-107	*Globicatella sp.*	HIA	Aerobic	Health
COT-036	*LachnospiraceaeXIVa [G-3] sp.*	HIA	Anaerobic	PD
COT-044	*Peptococcus sp.* COT-044†	ASA	Anaerobic	PD
COT-064	*Filifactor sp. ZP078*	HIA	Anaerobic	
COT-227	*Peptostreptococcus sp.*	HIA	Anaerobic	
COT-388	*Clostridiales III [G-3] sp.*	HIA	Anaerobic	PD
COT-033	Peptostreptococcus sp.*	CBA H + M	Anaerobic	
COT-016	Neisseria animaloris*	CBA	Aerobic	
COT-192	Porphyromonas sp. IJD1952*	CBA H + M	Anaerobic	
COT-108	Porphyromonas canoris*	CBA H + M	Anaerobic	
COT-052	Porphyromonas gulae II*	CBA H + M	Anaerobic	
COT-080	Pasteurellaceae [G-2] sp.*	CBA	Aerobic	Health
COT-029	Tissierella sp. COT-029	Not isolated	Anaerobic	
COT-306	*Chloroflexi* sp. COT-306	Not isolated	Aerobic	PD
COT-280	*Conchiformibius steedae* COT-280	Not isolated	Aerobic	

The canine oral taxon number relates to taxonomy assigned to species identified as part of a culture independent survey of the canine oral microbiome (Dewhirst et al., [9]). †denotes the requirement of helper strain *P. acnes* and *denotes previously isolated species that were targeted for a study of intra-species diversity.

The majority of target species were able to grow in isolation on heart infusion agar. Some media sustained high levels of certain target species in mixed culture but did not support the growth of these species in isolation. This suggests complementation of missing essential nutrients for these species via secondary metabolites produced by co-cultured species. We found that *Peptococcus* sp. COT-044 and *Odoribacter denticanis* required artificial saliva agar and the use of a *P. acnes* helper strain, respectively (Table 3).

Three species would not grow in isolation on any medium tested or with the helper strain *P. acnes*, although they were significantly enriched by the selection process used. These uncultivated species belong to 3 different phylogenetic families (Clostridiales, Chloroflexi and Neisseriaceae). It is presumed that other species present in the mixed cultures were acting as helper species for these (shaded in grey in Table 3).

## Conclusions

Using a combination of generic media in a culture based survey and a target screen using optimized media it has been possible to isolate species from most clades in the canine oral microbiome including a number that had never previously been grown in the laboratory. This validates the utility of the screening procedure and focusses future efforts for other as yet uncultivated species on identifying the correct media for growth.

## Competing interests

We have the following interests; this work was funded by Mars Petcare UK, the employer of the authors of this paper.

## Authors’ contributions

IJD & SH contributed towards the study by making substantial contributions to conception, experimental design, data analysis, data interpretation and in drafting the manuscript. CB, IM & AH contributed towards the study by making substantial contributions to experimental design, data analysis, data interpretation and in drafting the manuscript. All authors read and approved the final manuscript.

## Supplementary Material

Additional file 1 Examples of qPCR data demonstrating the drop in Ct values as the relative prevalence of three target species (COT-064, COT-107 & COT-227) increased through the screening process. Click here for file

Additional file 2 Examples of qPCR plots gained during isolation of three target species (COT-064, COT-107 & COT-227). Click here for file

Additional file 3 16S rRNA sequence generated to confirm the identity of three target species as examples of species isolated in this work. Click here for file
