# Supplementary material for: The Unculturables: targeted isolation of bacterial species associated with canine periodontal health or disease from dental plaque
Source: BMC Microbiol. 2014 Aug 1;14:196. doi: 10.1186/1471-2180-14-196 (PMC4236526; doi:10.1186/1471-2180-14-196)
Supplement: Additional file 2 — Examples of qPCR plots gained during isolation of three target species (COT-064, COT-107 & COT-227). [file 1471-2180-14-196-S2.docx]

# Supplementary Data 2. Examples of qPCR plots gained during isolation of three target species (COT-064, COT-107 & COT-227).

# Isolation of Canine Oral Taxon-064


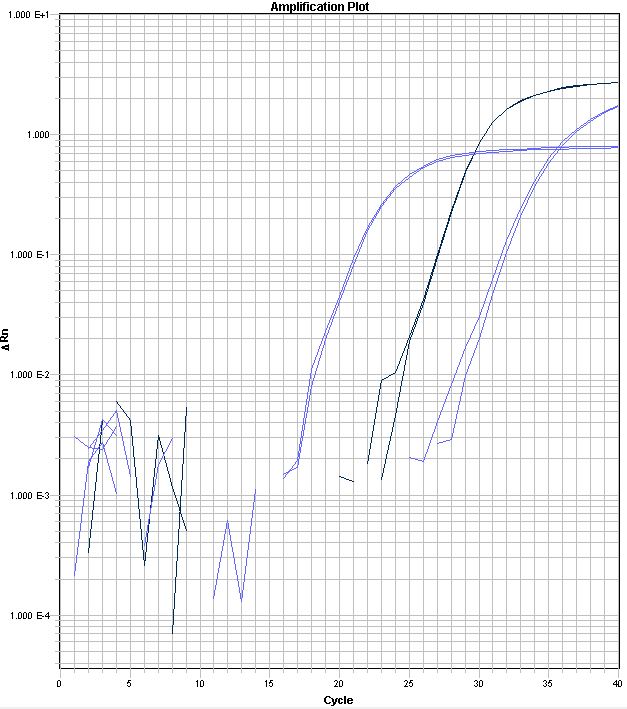


Chart 1: A qPCR plot demonstrating the identification of COT-064 and COT-277 in plaque sample number 9. Key from Left to right: blue line = uniB amplicon, Black = COT-064, purple = COT-227


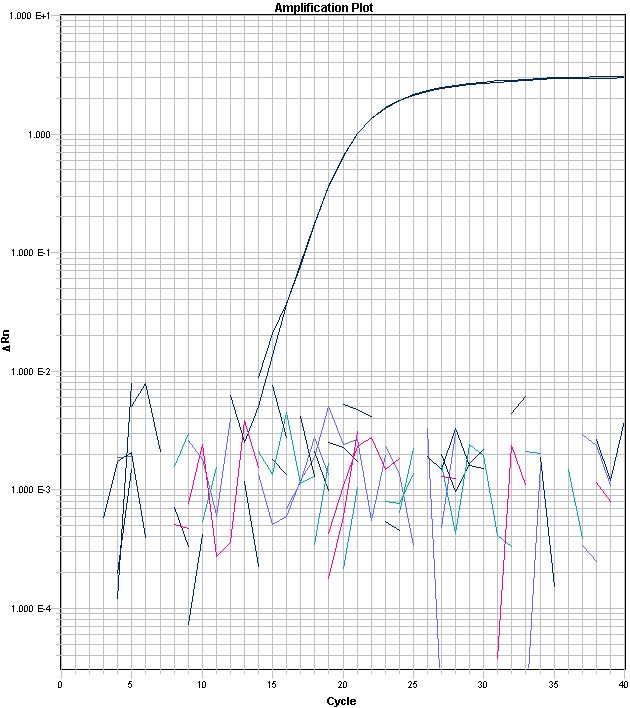


Chart 2: Example of a screen of a mixed colony spot for multiple target COTs. Key Black = COT-064, undetected taxa included = COT-006, COT-019, COT-021, COT-024, COT-227


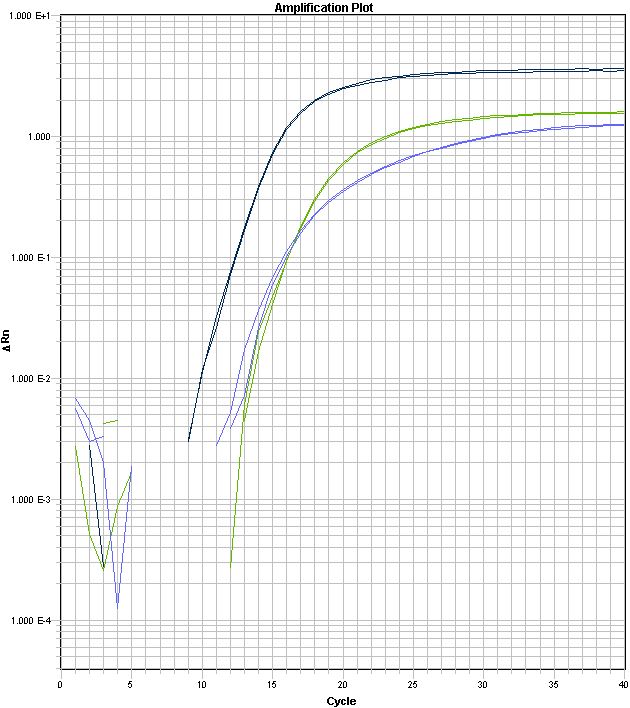


Chart 3: An example of the second round of screening of target spot still containing mixed species. Key left to right: Black= COT-064 , Blue=UniB and Green= COT109. Note variations in efficiency of each primer set hence COT-064 amplicon appears prior to the universal primer set. UniB efficiency is compromised in order to amplify 16S rDNA froma as great a range of taxa as possible.


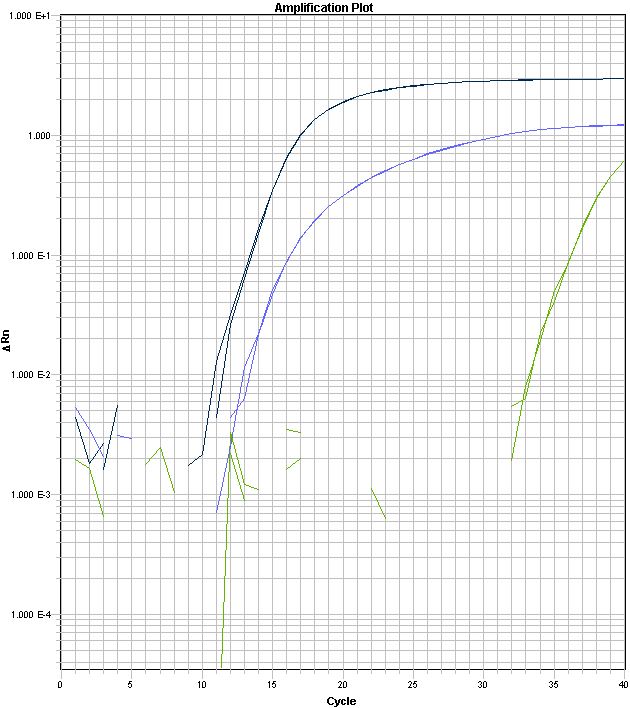


Chart 4: Further purification of COT-064 from a spot primarily containing two species Key left to right: Black= COT-064 , Blue=UniB and Green= COT109


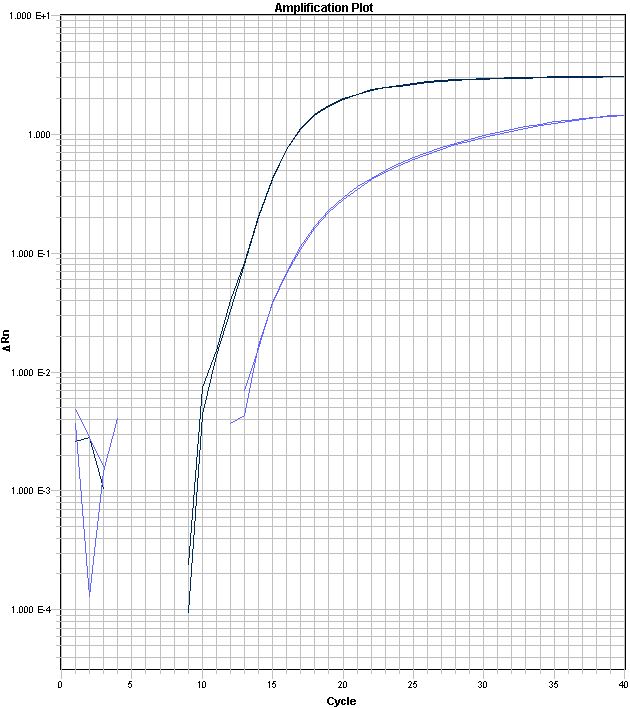


Chart 5: Screening a single colony morphology restreak with COT-064 probe set. Key left to right: Black= COT-064 and Blue=UniB. This restreak represented a pure isolate of COT-064.

# Isolation of COT-107


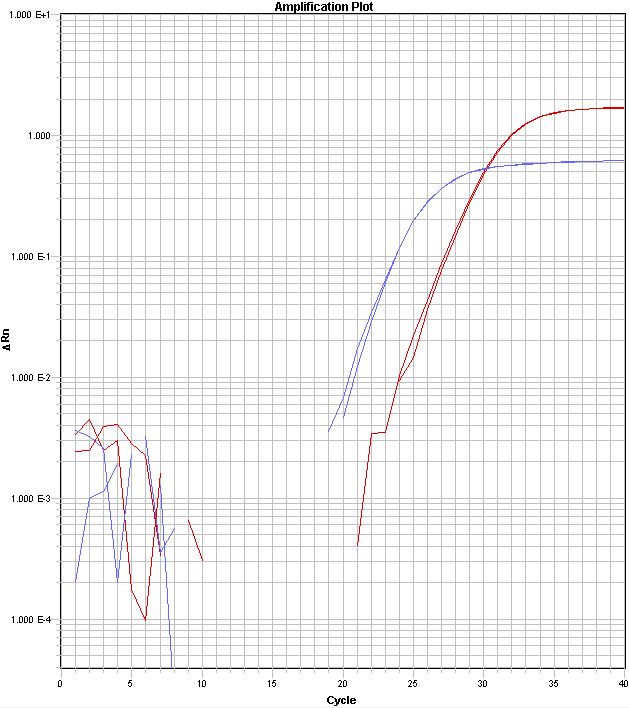


Chart 6: Identification of COT-107 in plaque sample number 10. Key left to right: Blue line= UniB and Red line = COT-107 amplicon.


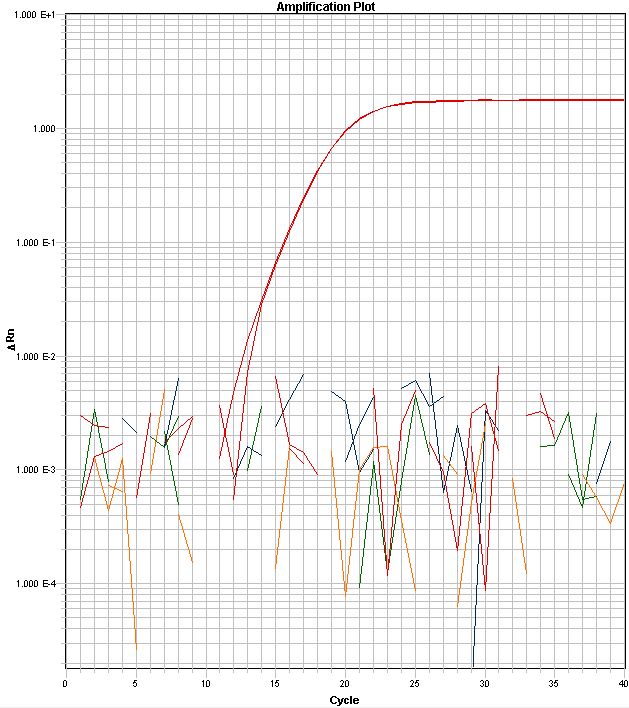


Chart 7: An example of a screen of a mixed colony spot from plaque sample 10 for multiple target COTs. Key left to right, red line COT107, undetected = COT019, COT024, COT030, COT044


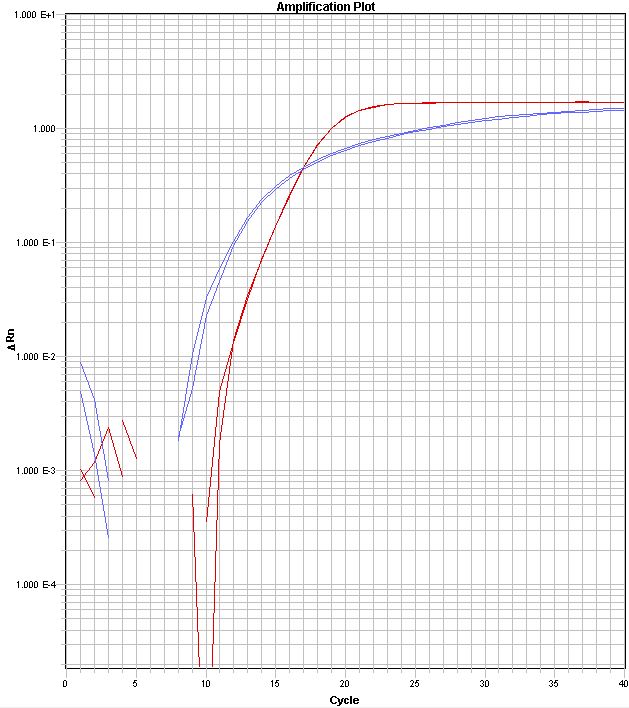


Chart 8: An example of the second round of screening of target spots still containing mixed species. Key left to right: Blue line = Unib and Red line= COT-107


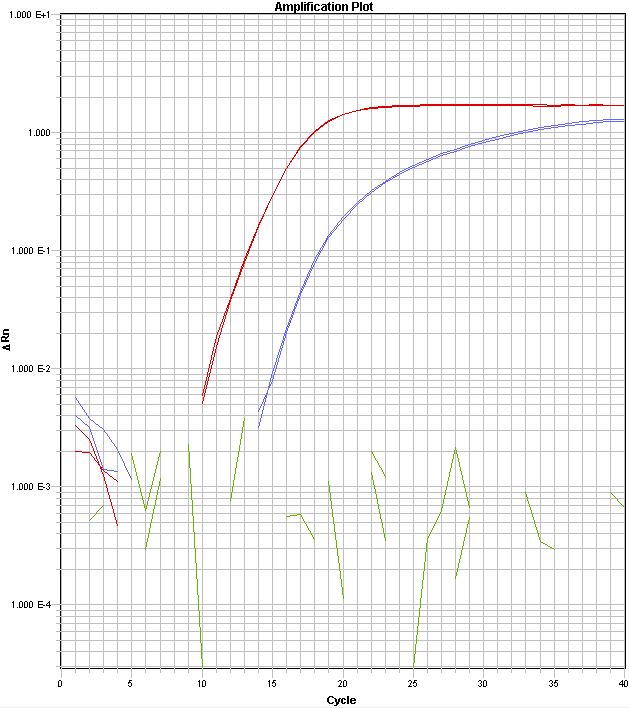


Chart 9: Screening a single colony morphology restreak. Key left to right: Red line= COT-107, blue = Unib = blue and green =undetectable COT-109. This restreak represented a pure isolate of COT-107.

# Isolation of COT-227


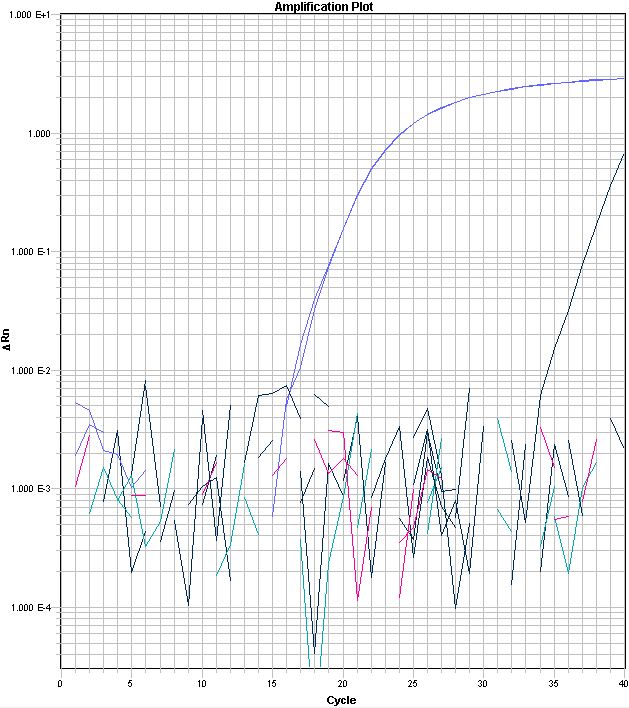


Chart 10: An example of a screen of a mixed colony spot for multiple target COTs. Key left to right: purple = COT-227, black =COT- 064, undetected = COT006, COT019, COT021, COT024


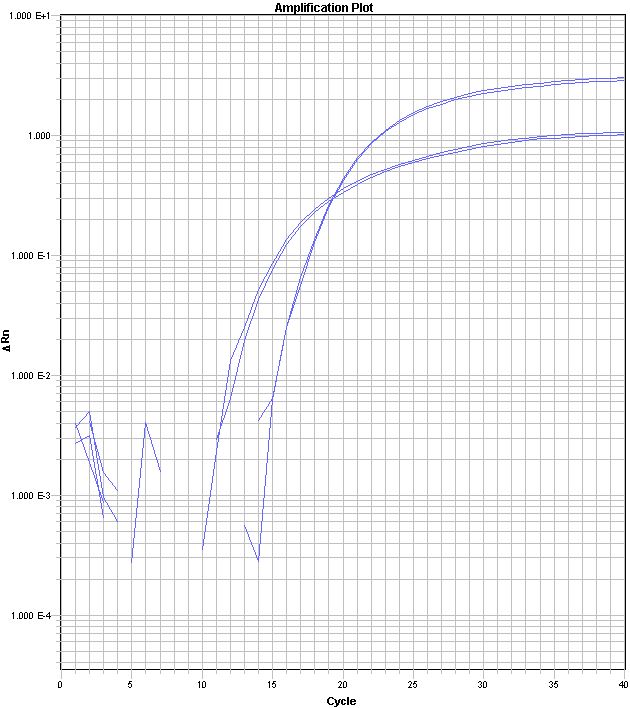


Chart 11. An example of the third round of screening of a target spot. Key left to right: blue = Unib, and purple =COT- 227


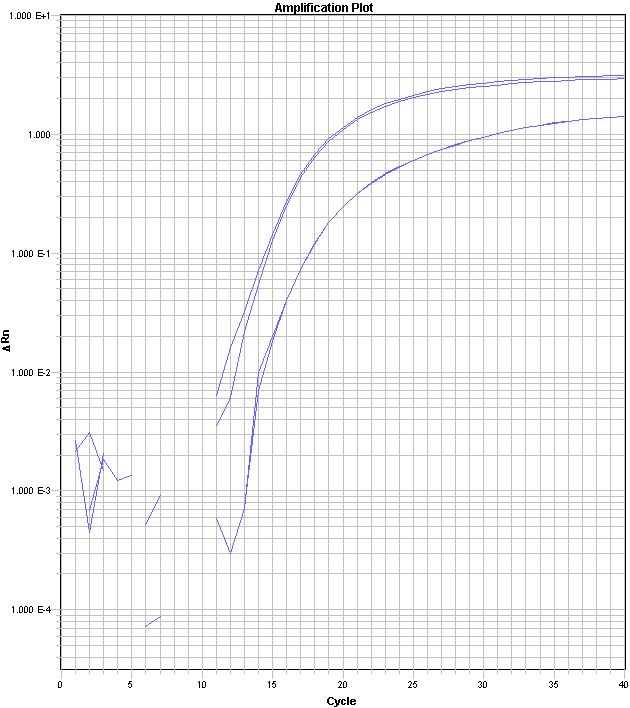


Chart 12. Screening a single colony morphology restreak with COT-227 probe set. Key left to right: purple = COT-227, blue = Unib. This restreak represented a pure isolate of COT-227
